# *Toxoplasma gondii* seroprevalence varies by cat breed

**DOI:** 10.1371/journal.pone.0184659

**Published:** 2017-09-08

**Authors:** Kärt Must, Marjo K. Hytönen, Toomas Orro, Hannes Lohi, Pikka Jokelainen

**Affiliations:** 1 Institute of Veterinary Medicine and Animal Sciences, Estonian University of Life Sciences, Tartu, Estonia; 2 Research Programs Unit, Molecular Neurology, University of Helsinki, Helsinki, Finland; 3 Folkhälsan Institute of Genetics, Helsinki, Finland; 4 Department of Veterinary Biosciences, Faculty of Veterinary Medicine, University of Helsinki, Helsinki, Finland; 5 Statens Serum Institut, Copenhagen, Denmark; Universita degli Studi di Parma, ITALY

## Abstract

*Toxoplasma gondii* is a widespread zoonotic parasite that is relevant for veterinary and public health. The domestic cat, the definitive host species with the largest worldwide population, has become evolutionarily and epidemiologically the most important host of *T*. *gondii*. The outcome of *T*. *gondii* infection is influenced by congenital and acquired host characteristics. We detected differences in *T*. *gondii* seroprevalence by cat breed in our previous studies. The aims of this study were to estimate *T*. *gondii* seroprevalence in selected domestic cat breeds, and to evaluate whether being of a certain breed is associated with *T*. *gondii* seropositivity, when the age and lifestyle of the cat are taken into account. The studied breeds were the Birman, British Shorthair, Burmese, Korat, Norwegian Forest Cat, Ocicat, Persian, and Siamese. Plasma samples were analyzed for the presence of immunoglobulin G antibodies against *T*. *gondii* with a commercial direct agglutination test at dilution 1:40. The samples were accompanied by owner-completed questionnaires that provided background data on the cats. Overall, 41.12% of the 1121 cats tested seropositive, and the seroprevalence increased with age. The Burmese had the lowest seroprevalence (18.82%) and the Persian had the highest (60.00%). According to the final multivariable logistic regression model, the odds to test seropositive were four to seven times higher in Birmans, Ocicats, Norwegian Forest Cats, and Persians when compared with the Burmese, while older age and receiving raw meat were also risk factors for *T*. *gondii* seropositivity. This study showed that *T*. *gondii* seroprevalence varies by cat breed and identified being of certain breeds, older age, and receiving raw meat as risk factors for seropositivity.

## Introduction

*Toxoplasma gondii* is an intracellular protozoan parasite with a broad host range [1]. Sexual stages of *T*. *gondii* develop only in the intestine of felids, which are able to shed *T*. *gondii* oocysts into the environment if infected with the parasite [1]. In the intestinal epithelium of felids, recombination can occur and yield genetically divergent *T*. *gondii* strains [2]. Domestic cats (*Felis catus*) are the only domestic animals that can be definitive hosts for *T*. *gondii*. As the definitive host species with the largest worldwide population, the domestic cat has become evolutionarily and epidemiologically the most important host of the parasite [2].

In the majority of host species, including humans (*Homo sapiens*) and domestic cats, most *T*. *gondii* infections are subclinical and chronic, and can be diagnosed indirectly by detecting specific anti-*T*. *gondii* antibodies with serologic tests [1]. However, this zoonotic parasite causes disease and mortality in many of its hosts, including humans, cats, other domestic animals, and wildlife, which makes it relevant for both veterinary and public health [1].

The strain of the parasite, the infection dose and route, as well as congenital and acquired host characteristics, can affect the outcome of *T*. *gondii* infection [3–7]. Host-parasite relationships and coevolution have influenced the balance between the hosts and the parasite, susceptibility of the hosts, and virulence of the parasite [1, 2]. Host species [8–11], breeds [12, 13], strains [14–17], and families [18] display apparent differences in their susceptibility to the infection. The differences appear genetic [5, 6] and may be explained by differences in the immune responses elicited [19, 20].

Domestic cats of certain breeds appear to be more susceptible to infections and diseases than others. A predisposition to the development of feline infectious peritonitis, caused by certain strains of feline coronavirus, was found in Abyssinians, Bengals, Birmans, Himalayans, Ragdolls, and Rexes [21], and specific candidate genes associated with the susceptibility have been identified in Birmans [22]. Devon Rexes have been suggested to be predisposed to skin colonization by fungi of the genus *Malassezia* [23].

We detected differences by breed in *T*. *gondii* seroprevalence in domestic cats in our previous studies [24, 25]. The limited number of cats included per breed did not allow further investigation of the observation in those studies, both of which identified older age and lifestyle as risk factors for seropositivity. We designed this study to investigate the observation; our hypothesis was that being of a certain domestic cat breed would be a risk factor or a protective factor for *T*. *gondii* seropositivity. The aims of this study were to estimate *T*. *gondii* seroprevalence in selected domestic cat breeds and to test the hypothesis, taking into account the age and lifestyle of the cat.

## Materials and methods

### Ethics statement

No animals were sampled primarily for this study. The plasma samples were collected earlier and stored in the Finnish feline biobank. They were utilized under the owners’ consent for research use (ethical permit ESAVI-2010-0392/Ym-23). All data were handled confidentially, and the results are presented so that individual cats cannot be identified.

### Selection and exclusion of breeds

The selection of breeds was based on the findings in our previous studies in Finland and Estonia [24, 25]. We included breeds with a seroprevalence estimate that differed statistically (P < 0.05) from the prevalence in all other purebred cats or the overall prevalence that included also non-purebred cats, and breed pairs that had statistically different (P < 0.05) seroprevalences, in either of the previous studies. The breeds that fulfilled the inclusion criteria were the Birman, British Shorthair, Burmese, Korat, Norwegian Forest Cat, Ocicat, Persian, Scottish Fold, and Siamese. The Scottish Fold was not included, however, because there were no available samples from cats of this breed.

### Samples

This study utilized plasma samples separated from whole blood samples collected for the Finnish feline biobank by February 2015 from the Birman, British Shorthair, Burmese, Korat, Norwegian Forest Cat, Ocicat, Persian, and Siamese. Most cats were represented by a single sample. Two samples were available and analyzed from 156 individual cats. We also included test results from 74 cats from our previous study that included samples from the same biobank [24]. The final sample size was 1121 cats.

The samples were stored at -20°C and coded for blinded analyses.

### Questionnaire data

Each sample had been accompanied by a questionnaire completed by the owners of the cats. In addition to the breed, the questionnaires provided information on the date or year when the cat was born and the date the sample was taken. From these, the age of the cat in years at the time of sampling was calculated. Data on gender, whether the cat received raw meat, and whether the cat had outdoor access were also requested through the questionnaire. The background information of the cats is summarized in S1 Table. Data were missing for some cats: age in years for 22 cats, age group for 18 cats, gender for one cat, information about receiving raw meat for 30 cats, and information about outdoor access for 27 cats.

### Serology

We tested the plasma samples for the presence of anti-*T*. *gondii* immunoglobulin G antibodies with a commercial direct agglutination test (Toxo-Screen DA, bioMérieux, Marcy-l’Étoile, France) according to the manufacturer’s instructions. The method has not been validated for analysis of feline samples, but it has been used for detecting antibodies against *T*. *gondii* in cats in several studies [1, 24, 25]. In this method, possible immunoglobulin M antibodies are denatured by 2-mercaptoethanol. The samples were diluted 1:40, and samples that tested positive were defined as seropositive. All plates contained the positive and negative controls provided with the kit in two dilutions (1:40 and 1:4000), and antigen control. We read the results after 18 hours of incubation. Our previous study [24] used the same method and cut-off for determining seropositivity, thus allowing for inclusion of the results of 74 cats from that study.

### Statistical analyses

Using 50% as the expected seroprevalence, the sample sizes available from the breeds Birman and Norwegian Forest Cat were evaluated as sufficient for obtaining an estimate of the breed-level seroprevalence at 90% confidence level, while the sample sizes from other breeds were limited [26].

We report a seroprevalence estimate for each of the breeds. In addition, we categorized the cats by their breed as long-haired (Birmans, Norwegian Forest Cats, and Persians) and short-haired (British Shorthairs, Burmese, Korats, Ocicats, and Siamese). Confidence intervals (CI) for the prevalence estimates were computed using mid-P exact, and the differences were evaluated using two-by-two tables and mid-P exact of the open source software Open Epi 3.01 [26]. The P-values for all analyses are reported. There were multiple comparisons and the P-values should be interpreted with caution: we considered P-values < 0.001 as statistically significant.

Stata 13.1 (StataCorp, College Station, TX, USA) was used to generate logistic regression models to evaluate the association of five variables with the dichotomous outcome, *T*. *gondii* seropositivity. Age of the cat was evaluated as a continuous variable in years, categorized to 0-, 1-, 2-, 3-, 4–6- and 7–19-year-olds based on the age distribution of the cats, and dichotomized to two age groups: kitten if less than one year old, or adult if at least one year old. We did not assign age group for five cats from which two samples were available and which had been kittens at the first and adults at the second sampling. The other variables were breed, gender, whether the cat was receiving raw meat, and whether the cat had outdoor access.

Univariable analyses were used to evaluate the variables separately. Each variable was evaluated for each breed as well as for all the cats, regardless of breed. Age of the cat was evaluated as dichotomous variable for each of the breeds, and in addition as continuous variable for all the cats, regardless of breed.

Multivariable logistic regression models were built by offering all the variables to the model followed by backward elimination of variables with P-values ≥ 0.05 starting from the highest P-value, taken the variable did not act as a confounder. The predictive power of the models is presented as area under the receiver operating characteristic curve.

A model was first built for each breed as well as for all the cats, regardless of breed. In these models, the age of the cat was included as a dichotomous variable (age group).

The final model included the breeds as dummy variables and used the age categories. Eight different versions were built to evaluate all the possible comparisons between breeds. The final model had the breed with the lowest seroprevalence as the reference breed.

## Results

The overall seroprevalence of anti-*T*. *gondii* immunoglobulin G antibodies was 41.12% (95% CI 38.28–44.03, Table 1). No seroconversions or seroreversions were detected in the cats from which two samples were tested. The questionnaire data and test result for each individual cat are presented in S6 Table.

**Table 1:** *Toxoplasma gondii* seroprevalence in domestic cats of eight breeds.

Breed	N	N seropositive	% seropositive	95% confidence interval (mid-P exact)
**Persian**	60	36	60.00	47.35–71.44
**Norwegian Forest Cat**	343	160	46.65	41.43–51.93
**Birman**	281	127	45.20	39.48–51.04
**Ocicat**	88	38	43.18	33.33–53.60
**Siamese**	43	15	34.88	21.37–49.88
**British Shorthair**	107	36	33.64	25.38–43.04
**Korat**	114	33	28.95	21.39–37.88
**Burmese**	85	16	18.82	11.83–28.52
**Total**	1121	461	41.12	38.28–44.03

**Fig 1:**
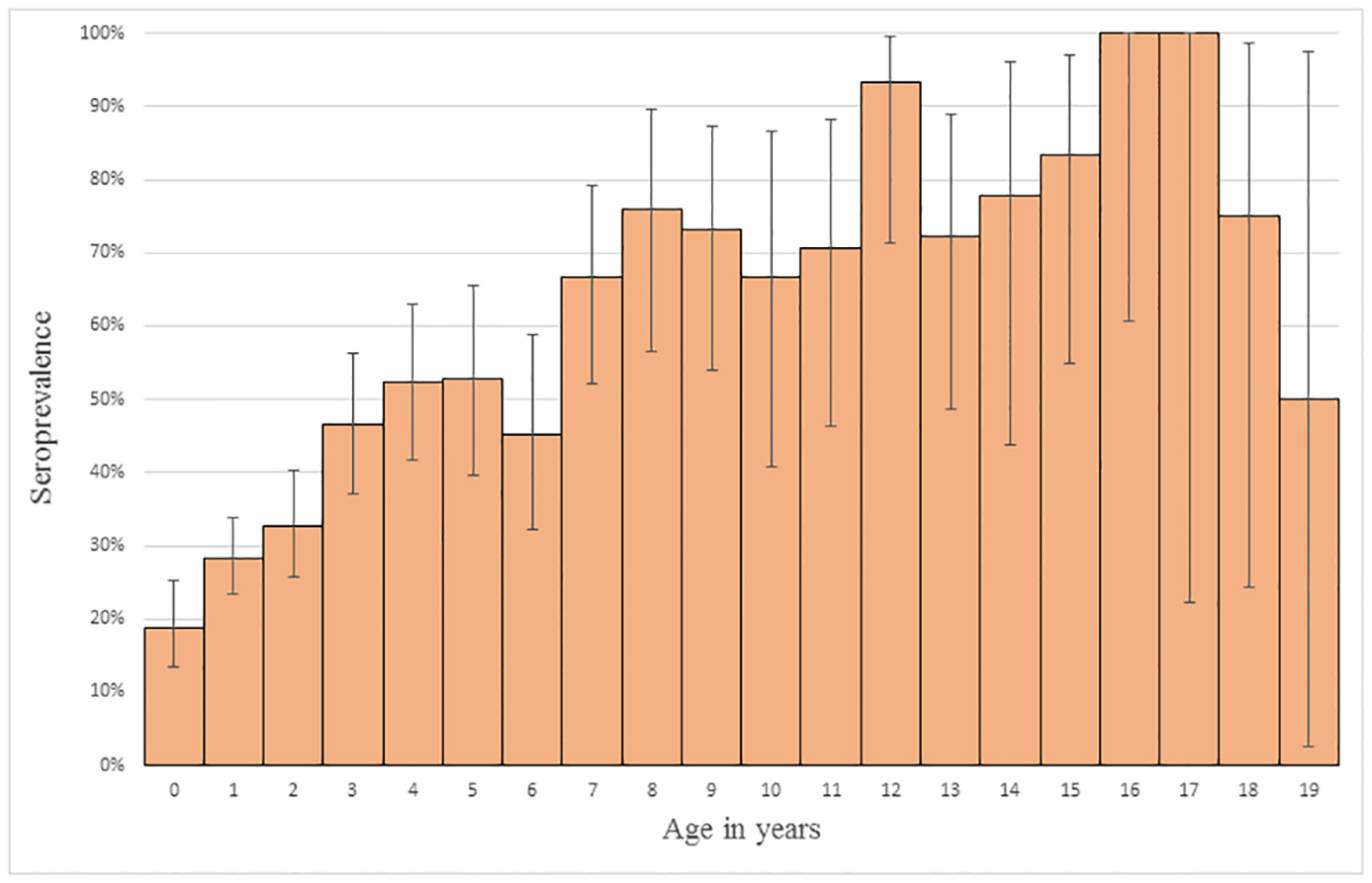
Histogram showing the increase in *Toxoplasma gondii* seroprevalence with the age of the cats. The proportion of cats testing seropositive and the 95% confidence interval are shown.

The seroprevalence increased with age (Fig 1). The seroprevalence in adult cats was 45.20% (95% CI 42.04–48.40), which was higher (P < 0.001) than the seroprevalence in kittens, 18.79% (95% CI 13.52–25.47).

Burmese had the lowest seroprevalence (18.82%) and Persians had the highest (60.00%). P-values of the two-by-two tables comparing two breeds at the time are shown in S2 Table. Among the cats included in this study, the seroprevalence in cats of long-haired breeds was 47.2% (95% CI 43.5–51.0), which was higher (P < 0.001) than the seroprevalence in cats of short-haired breeds, 31.6% (95% CI 27.4–36.1). The odds of testing seropositive were 1.94 (95% CI 1.51–2.50) times higher among long-haired cats than in short-haired cats.

In the univariable logistic regression models generated for each breed separately (S3 Table), being an adult cat was a risk factor for *T*. *gondii* seropositivity for Birmans and Norwegian Forest Cats. Receiving raw meat was a risk factor for Birmans and Burmese. Having outdoor access was a risk factor for Norwegian Forest Cats. Being of male gender appeared as a protective factor for Birmans. The univariable analyses for all cats, regardless of breed (S3 Table), identified being an adult cat, receiving raw meat, and having outdoor access as risk factors for *T*. *gondii* seropositivity. The model using age as a continuous variable suggested that the odds to test seropositive would increase with a factor of 1.24 (95% CI 1.19–1.29) for each additional year.

In the multivariable logistic regression model built for Birmans, being an adult cat and receiving raw meat were risk factors, while being male was a protective factor (S4 Table). The model built for Norwegian Forest Cats included two risk factors: being an adult and having outdoor access. In the model built for Burmese, receiving raw meat was the only risk factor. The multivariable model built for all cats, regardless of breed (S4 Table), included being an adult cat and receiving raw meat as risk factors.

The final multivariable logistic regression model for *T*. *gondii* seropositivity included the breeds as dummy variables (the Burmese as the reference breed), age as a categorized variable (< 1 year old as the reference category), and whether the cat received raw meat or not as a dichotomous variable (Table 2). The odds to test seropositive were four to seven times higher in cats of breeds Birman, Ocicat, Norwegian Forest Cat and Persian when compared with the Burmese, while the model also confirmed that older age and receiving raw meat were relevant risk factors for *T*. *gondii* seropositivity. Age was identified as a confounder. The results from the models with different breeds as the reference are shown in supplementary tables (S5 Table).

**Table 2:** The final multivariable logistic regression model predicting *Toxoplasma gondii* seropositivity in 1121 domestic cats of eight breeds.

	Odds ratio	95% confidence interval	P-value
**Burmese (n = 85)**	Reference		
**Birman (n = 281)**	4.16	2.12–8.19	0.000
**British Shorthair (n = 107)**	3.39	1.57–7.30	0.002
**Korat (n = 114)**	2.03	0.94–4.42	0.073
**Norwegian Forest Cat (n = 343)**	4.66	2.39–9.08	0.000
**Ocicat (n = 88)**	4.26	1.94–9.38	0.000
**Persian (n = 60)**	6.99	2.97–16.45	0.000
**Siamese (n = 43)**	2.57	1.01–6.53	0.047
**< 1 year old (n = 165)**	Reference		
**1-year-old (n = 286)**	1.74	1.07–2.83	0.025
**2-year-old (n = 159)**	2.02	1.19–3.43	0.009
**3-year-old (n = 103)**	4.19	2.36–7.45	0.000
**4–6-year-old (n = 190)**	4.91	2.97–8.13	0.000
**7–19-year-old (n = 196)**	16.08	9.36–27.61	0.000
**Not receiving raw meat (n = 178)**	Reference		
**Receiving raw meat (n = 913)**	2.46	1.58–3.82	0.000

Area under the receiver operating characteristic curve = 0.7587

## Discussion

This study showed that *T*. *gondii* seroprevalence varies considerably by cat breed, and the observation that long-haired cats had higher odds to test seropositive is intriguing. Being of certain breeds was confirmed to be a risk factor for testing *T*. *gondii* seropositive also when the age and lifestyle of the cats were taken into account. With this study design, it remains unknown from where the differences by breed arose; they might be due to genetic susceptibility or differences in the type, strength, or length of the measurable humoral response. Cats of any of the breeds included in this study cannot be considered safe from *T*. *gondii*.

This study was limited to selected breeds and does not represent all purebred cats nor the local population of cats. The percentage of cats that tested positive for anti-*T*. *gondii* antibodies was lower than the *T*. *gondii* seroprevalence estimates from Finland and Estonia (P < 0.01 and P < 0.001, respectively) [24, 25]. A similar proportion of kittens was included in this study as in the Finnish study [24], whereas the larger proportion of kittens included in this study than in the Estonian study [25] could partly explain that difference. All these estimates of seroprevalence are conservative and likely underestimate *T*. *gondii* infection prevalence, because the method used is indirect and detects only immunoglobulin G antibodies. Recently infected cats have not produced these antibodies yet and appear seronegative. Moreover, using a single dilution, we expected few cats with high antibody titers to test seronegative because of the prozone phenomenon [24].

The Finnish feline biobank, which was utilized in this study, is based on voluntary contributions. The cat owners who had contributed to the biobank might keep their cats differently than cat owners who did not participate. Moreover, cats that are difficult to handle at a veterinary clinic may be less likely to be sampled. Thus, the results of this study may represent mainly co-operative cats whose owners were interested in participating in the feline biobank effort. In addition, different types of owners might be more likely to choose cats of particular breeds, which can influence the lifestyle of cats of a certain breed. The background information for the cats was reported by the owners, and there may be recall bias.

How cats are kept varies around the world, and *T*. *gondii* infection pressure varies between and within countries. In Finland, a geographical north-south gradient in *T*. *gondii* seroprevalence has been described in Eurasian lynx (*Lynx lynx*) [27], and in moose (*Alces alces*) and domestic sheep (*Ovis aries*) [28]. The lynxes that originated from the south-west of Finland had a significantly higher *T*. *gondii* seroprevalence than the lynxes from north-east [27]. Similarly, the seroprevalence in moose and sheep was higher in south-western and lower in northern parts of Finland [28]. It is unknown whether the gradient in *T*. *gondii* prevalence is present also in small prey that domestic cats that are allowed to hunt could catch, but the seroprevalence in cats living near the capital city, located in the south, was similar to that in cats from other parts of the country [24]. For each cat included in this study, in addition to breed, we had data on the known major risk factors for *T*. *gondii* seropositivity: age, whether the cat was receiving raw meat, and whether the cat had outdoor access. Because the infection is usually acquired and seroprevalence increases with age (as shown also in this study), these risk factors were relevant to include in the analyses. In particular, raw meat given to the cats by the owners is a major lifestyle risk factor for *T*. *gondii* seropositivity in Finland [24]. In this study, in addition to the breed of the cat, the age of the cat and whether it was receiving raw meat were statistically significant variables in the final multivariable model for *T*. *gondii* seropositivity. The type and origin of the raw meat given to the cats were, unfortunately, unknown.

Felids are the only known definitive hosts of *T*. *gondii*—the only hosts that can shed *T*. *gondii* oocysts. Mammals, including humans, and birds can acquire *T*. *gondii* infection by ingesting sporulated oocysts, which have been shed in unsporulated form by infected felids. The only wild felid living in Finland is the Eurasian lynx (*Lynx lynx*), which has not been shown to shed *T*. *gondii* oocysts [27]. The domestic cat is considered to be the most important host species of *T*. *gondii* evolutionarily and also epidemiologically for example in regions where domestic cats outnumber the other definitive hosts [2, 24, 27]. Most seropositive cats are considered to have previously shed oocysts in their feces [1]. From serology results, we can thus retrospectively conclude that a high proportion of cats of a breed with high *T*. *gondii* seroprevalence has shed *T*. *gondii* oocysts, while the proportional potential contribution to the environmental oocyst contamination by cats of a breed with lower seroprevalence has been smaller. It is unknown whether shedding of *T*. *gondii* oocysts (length of the shedding period, number of oocysts shed) differs by domestic cat breed.

*Toxoplasma gondii* seroprevalence varied considerably by breed, but seropositive cats were detected in all the breeds studied and the proportion of seropositive cats was overall high. Cats of any of the breeds included in this study cannot be considered being safe from *T*. *gondii*. Eating raw meat, the risky lifestyle factor that was also associated with seropositivity, is avoidable, and protecting cats of all breeds from *T*. *gondii* infection should be promoted.

If cats less susceptible to *T*. *gondii* infection could be selected for breeding, it could have a major impact. Preferring such cats could potentially decrease the risk of infection for other hosts, including humans, by limiting the production of *T*. *gondii* oocysts. Furthermore, as *T*. *gondii* can cause severe disease and mortality in domestic cats [24, 29], decreasing susceptibility to the parasite would also be beneficial for feline welfare and health.

## Supporting information

S1 Table Age, gender, and lifestyle of the cats included in this study, by breed. (PDF)

S2 Table P-values (mid-P exact) of two-by-two table comparisons of proportion of cats testing *T*. *gondii* seropositive, by breed. (PDF)

S3 Table Univariable logistic regression models for *Toxoplasma gondii* seropositivity for cats of eight breeds separately as well as for all cats, regardless of breed. (PDF)

S4 Table Multivariable logistic regression models for *Toxoplasma gondii* seropositivity for cats of eight breeds separately as well as for all cats, regardless of breed. (PDF)

S5 Table The multivariable logistic regression models for *Toxoplasma gondii* seropositivity in 1121 cats of eight breeds, using each of the breeds as the reference breed. (PDF)

S6 Table The questionnaire data and test result for each individual cat. (XLSX)
