# Supplementary material for: Toxoplasma gondii seroprevalence varies by cat breed
Source: PLoS One. 2017 Sep 8;12(9):e0184659. doi: 10.1371/journal.pone.0184659 (PMC5590984; doi:10.1371/journal.pone.0184659)
Supplement: S1 Table — (PDF) [file pone.0184659.s001.pdf]

S1 Table. Age, gender, and lifestyle of the cats included in this study, by breed.

	n	Mean age (years)	Median age (full years)	% < 1 year old	% male	% receiving raw meat	% having outdoor access
**Birman**	281	3.52	2	15.10	41.28	86.45	64.62
**British Shorthair**	107	2.34	1	28.85	37.38	89.62	36.79
**Burmese**	85	3.74	2	14.29	37.65	67.50	60.00
**Korat**	114	4.64	3	9.65	52.63	41.44	30.09
**Norwegian Forest Cat**	343	3.59	2	17.21	44.44	91.76	77.74
**Ocicat**	88	2.94	2	9.41	51.14	97.62	56.32
**Persian**	60	3.59	2	6.78	43.33	92.59	58.82
**Siamese**	43	3.83	2.5	0.00	32.56	88.37	39.53
**Total**	**1121**	**3.53**	**2**	**14.96**	**43.30**	**83.68**	**60.15**
