# Supplementary material for: Toxoplasma gondii seroprevalence varies by cat breed
Source: PLoS One. 2017 Sep 8;12(9):e0184659. doi: 10.1371/journal.pone.0184659 (PMC5590984; doi:10.1371/journal.pone.0184659)
Supplement: S2 Table — (PDF) [file pone.0184659.s002.pdf]

S2 Table. P-values (mid-P exact) of two-by-two table comparisons of proportion of cats testing *T. gondii* seropositive, by breed.

**Breed** (seroprevalence %)	**Norwegian Forest Cat** (46.65)	**Birman** (45.20)	**Ocicat** (43.18)	**Siamese** (34.88)	**British Shorthair** (33.64)	**Korat** (28.95)	**Burmese** (18.82)
**Persian** (60.00)	0.058	0.039	0.047	0.013	0.001	0.000	0.000
**Norwegian Forest Cat** (46.65)		0.718	0.564	0.148	0.018	0.001	0.000
**Birman** (45.20)			0.744	0.209	0.209	0.003	0.000
**Ocicat** (43.18)				0.373	0.177	0.038	0.001
**Siamese** (34.88)					0.881	0.477	0.053
**British Shorthair** (33.64)						0.456	0.022
**Korat** (28.95)							0.104
