# Supplementary material for: Toxoplasma gondii seroprevalence varies by cat breed
Source: PLoS One. 2017 Sep 8;12(9):e0184659. doi: 10.1371/journal.pone.0184659 (PMC5590984; doi:10.1371/journal.pone.0184659)
Supplement: S3 Table — (PDF) [file pone.0184659.s003.pdf]

S3 Table. Univariable logistic regression models for *Toxoplasma gondii* seropositivity for cats of eight breeds separately as well as for all cats, regardless of breed. Odds ratio, 95% confidence interval, and P-value are shown.

**Breed**	**≥ 1 year old**	**Male gender**	**Receiving raw meat**	**Outdoor access**
**Birman**	4.18 (1.86–9.41), P=0.001	0.53 (0.33–0.87), P=0.012	2.91 (1.32–6.43), P=0.008	1.23 (0.75–2.02), P=0.416
**British Shorthair**	2.58 (0.94–7.07), P=0.066	1.10 (0.48–2.52), P=0.819	1.42 (0.35–5.71), P=0.622	0.80 (0.34–1.85), P=0.597
**Burmese**	0.66 (0.16–2.78), P=0.573	1.88 (0.63–5.62), P=0.262	8.75 (1.08–70.70), P=0.042	1 (0.32–3.15), P=1.000
**Korat**	1.94 (0.40-9.49), P=0.415	0.79 (0.35-1.78), P=0.572	1.78 (0.77-4.11), P=0.178	0.71 (0.28-1.78), P=0.460
**Norwegian Forest Cat**	4.33 (2.20–8.53), P=0.000	0.95 (0.62–1.45), P=0.809	1.39 (0.63–3.07), P=0.409	2.06 (1.20–3.53), P=0.008
**Ocicat**	1 (omitted)	1.61 (0.69–3.78), P=0.270	0.78 (0.05–12.95), P=0.864	1.65 (0.69–3.91), P=0.259
**Persian**	1 (omitted)	0.84 (0.30–2.39), P=0.750	1.38 (0.18–10.61), P=0.756	1.82 (0.58–5.71), P=0.306
**Siamese**	1 (omitted)	0.65 (0.16–2.60), P=0.548	1 (omitted)	0.25 (0.06–1.08), P=0.064
**All cats**	**3.57 (2.36–5.38), P=0.000**	**0.88 (0.69–1.12), P=0.290**	**2.58 (1.78–3.74), P=0.000**	**1.43 (1.11–1.83), P=0.005**
