# Supplementary material for: Toxoplasma gondii seroprevalence varies by cat breed
Source: PLoS One. 2017 Sep 8;12(9):e0184659. doi: 10.1371/journal.pone.0184659 (PMC5590984; doi:10.1371/journal.pone.0184659)
Supplement: S4 Table — (PDF) [file pone.0184659.s004.pdf]

S4 Table. Multivariable logistic regression models for *Toxoplasma gondii* seropositivity for cats of eight breeds separately as well as for all cats, regardless of breed. Odds ratio, 95% confidence interval, and P-value are shown.

**Breed**	**≥ 1 year old**	**Male gender**	**Receiving raw meat**	**Outdoor access**	**Area under the receiver operating characteristic curve**
**Birman**	3.73 (1.63–8.57), P=0.002	0.51 (0.31–0.86), P=0.011	2.58 (1.14–5.84), P=0.022		0.6620
**British Shorthair**					
**Burmese**			8.75 (1.08–70.70), P=0.042		0.6590
**Korat**					
**Norwegian Forest Cat**	4.14 (2.09–8.19), P=0.000			1.77 (1.01–3.10), P=0.046	0.6184
**Ocicat**					
**Persian**					
**Siamese**					
**All cats**	**3.65 (2.40–5.53), P= 0.000**		**2.74 (1.88–3.99), P=0.000**		**0.6188**
