# Supplementary material for: Toxoplasma gondii seroprevalence varies by cat breed
Source: PLoS One. 2017 Sep 8;12(9):e0184659. doi: 10.1371/journal.pone.0184659 (PMC5590984; doi:10.1371/journal.pone.0184659)
Supplement: S5 Table — (PDF) [file pone.0184659.s005.pdf]

S5 Tables. The multivariable logistic regression models for *Toxoplasma gondii* seropositivity in 1121 cats of eight breeds, using each of the breeds as the reference breed.

S5A Table. The multivariable logistic regression model for *Toxoplasma gondii* seropositivity in 1121 cats of eight breeds, using the Birman as the reference breed.

	**Odds ratio**	**95% confidence interval**	**P-value**
**Birman**	Reference		
**British Shorthair (n=107)**	0.81	0.48–1.36	0.434
**Burmese (n=85)**	0.24	0.122–0.472	0.000
**Korat (n=114)**	0.49	0.278–0.858	0.013
**Norwegian Forest Cat (n=343)**	1.12	0.783–1.60	0.537
**Ocicat (n=88)**	1.02	0.594–1.764	0.933
**Persian (n=60)**	1.68	0.885–3.182	0.113
**Siamese (n=43)**	0.61	0.292–1.302	0.205
**< 1 year old (n=165)**	Reference		
**1-year-old (n=286)**	1.74	1.07–2.83	0.025
**2-year-old (n=159)**	2.02	1.19–3.43	0.009
**3-year-old (n=103)**	4.19	2.36–7.45	0.000
**4–6-year-old (n=190)**	4.91	2.97–8.13	0.000
**7–19-year-old (n=196)**	16.08	9.36–27.61	0.000
**Not receiving raw meat (n=178)**	Reference		
**Receiving raw meat (n=913)**	2.46	1.58–3.82	0.000

Area under the receiver operating characteristic curve = 0.7587

S5B Table. The multivariable logistic regression model for *Toxoplasma gondii* seropositivity in 1121 cats of eight breeds, using the British Shorthair as the reference breed.

	**Odds ratio**	**95% confidence interval**	**P-value**
**British Shorthair (n=107)**	Reference		
**Birman (n=281)**	1.23	0.73–2.06	0.434
**Burmese (n=85)**	0.30	0.14–0.64	0.002
**Korat (n=114)**	0.60	0.31–1.17	0.136
**Norwegian Forest Cat (n=343)**	1.38	0.83–2.27	0.212
**Ocicat (n=88)**	1.26	0.66–2.41	0.488
**Persian (n=60)**	2.06	0.99–4.29	0.052
**Siamese (n=43)**	0.76	0.33–1.74	0.514
**< 1 year old (n=165)**	Reference		
**1-year-old (n=286)**	1.74	1.07–2.83	0.025
**2-year-old (n=159)**	2.02	1.19–3.43	0.009
**3-year-old (n=103)**	4.19	2.36–7.45	0.000
**4–6-year-old (n=190)**	4.91	2.97–8.13	0.000
**7–19-year-old (n=196)**	16.08	9.36–27.61	0.000
**Not receiving raw meat (n=178)**	Reference		
**Receiving raw meat (n=913)**	2.46	1.58–3.82	0.000

Area under the receiver operating characteristic curve = 0.7587

S5C Table. The multivariable logistic regression model for *Toxoplasma gondii* seropositivity in 1121 cats of eight breeds, using the Korat as the reference breed.

	**Odds ratio**	**95% confidence interval**	**P-value**
**Korat (n=114)**	Reference		
**Birman (n=281)**	2.05	1.17–3.59	0.013
**British Shorthair (n=107)**	1.66	0.85–3.26	0.136
**Burmese (n=85)**	0.49	0.23–1.07	0.073
**Norwegian Forest Cat (n=343)**	2.29	1.31–3.99	0.003
**Ocicat (n=88)**	2.10	1.04–4.21	0.038
**Persian (n=60)**	3.44	1.59–7.43	0.002
**Siamese (n=43)**	1.26	0.54–2.97	0.593
**< 1 year old (n=165)**	Reference		
**1-year-old (n=286)**	1.74	1.07–2.83	0.025
**2-year-old (n=159)**	2.02	1.19–3.43	0.009
**3-year-old (n=103)**	4.19	2.36–7.45	0.000
**4–6-year-old (n=190)**	4.91	2.97–8.13	0.000
**7–19-year-old (n=196)**	16.08	9.36–27.61	0.000
**Not receiving raw meat (n=178)**	Reference		
**Receiving raw meat (n=913)**	2.46	1.58–3.82	0.000

Area under the receiver operating characteristic curve = 0.7587

S5D Table. The multivariable logistic regression model for *Toxoplasma gondii* seropositivity in 1121 cats of eight breeds, using the Norwegian Forest Cat as the reference breed.

	**Odds ratio**	**95% confidence interval**	**P-value**
**Norwegian Forest Cat (n=343)**	Reference		
**Birman (n=281)**	0.89	0.63–1.28	0.537
**British Shorthair (n=107)**	0.73	0.44–1.20	0.212
**Burmese (n=85)**	0.21	0.11–0.42	0.000
**Korat (n=114)**	0.44	0.25–0.76	0.003
**Ocicat (n=88)**	0.91	0.54–1.55	0.742
**Persian (n=60)**	1.50	0.80–2.81	0.205
**Siamese (n=43)**	0.55	0.26–1.15	0.113
**< 1 year old (n=165)**	Reference		
**1-year-old (n=286)**	1.74	1.07–2.83	0.025
**2-year-old (n=159)**	2.02	1.19–3.43	0.009
**3-year-old (n=103)**	4.19	2.36–7.45	0.000
**4–6-year-old (n=190)**	4.91	2.97–8.13	0.000
**7–19-year-old (n=196)**	16.08	9.36–27.61	0.000
**Not receiving raw meat (n=178)**	Reference		
**Receiving raw meat (n=913)**	2.46	1.58–3.82	0.000

Area under the receiver operating characteristic curve = 0.7587

S5E Table. The multivariable logistic regression model for *Toxoplasma gondii* seropositivity in 1121 cats of eight breeds, using the Ocicat as the reference breed.

	**Odds ratio**	**95% confidence interval**	**P-value**
**Ocicat (n=88)**	Reference		
**Birman (n=281)**	0.98	0.57–1.68	0.933
**British Shorthair (n=107)**	0.79	0.41–1.52	0.488
**Burmese (n=85)**	0.23	0.11–0.52	0.000
**Korat (n=114)**	0.48	0.24–0.96	0.038
**Norwegian Forest Cat (n=343)**	1.09	0.64–1.86	0.742
**Persian (n=60)**	1.64	0.78–3.46	0.195
**Siamese (n=43)**	0.60	0.26–1.40	0.239
**< 1 year old (n=165)**	Reference		
**1-year-old (n=286)**	1.74	1.07–2.83	0.025
**2-year-old (n=159)**	2.02	1.19–3.43	0.009
**3-year-old (n=103)**	4.19	2.36–7.45	0.000
**4–6-year-old (n=190)**	4.91	2.97–8.13	0.000
**7–19-year-old (n=196)**	16.08	9.36–27.61	0.000
**Not receiving raw meat (n=178)**	Reference		
**Receiving raw meat (n=913)**	2.46	1.58–3.82	0.000

Area under the receiver operating characteristic curve = 0.7587

S5F Table. The multivariable logistic regression model for *Toxoplasma gondii* seropositivity in 1121 cats of eight breeds, using the Persian as the reference breed.

	**Odds ratio**	**95% confidence interval**	**P-value**
**Persian (n=60)**	Reference		
**Birman (n=281)**	0.60	0.31–1.13	0.113
**British Shorthair (n=107)**	0.48	0.23–1.01	0.052
**Burmese (n=85)**	0.14	0.06–0.34	0.000
**Korat (n=114)**	0.29	0.13–0.63	0002
**Norwegian Forest Cat (n=343)**	0.67	0.36–1.25	0.205
**Ocicat (n=88)**	0.61	0.29–1.29	0.195
**Siamese (n=43)**	0.37	0.15–0.91	0.031
**< 1 year old (n=165)**	Reference		
**1-year-old (n=286)**	1.74	1.07–2.83	0.025
**2-year-old (n=159)**	2.02	1.19–3.43	0.009
**3-year-old (n=103)**	4.19	2.36–7.45	0.000
**4–6-year-old (n=190)**	4.91	2.97–8.13	0.000
**7–19-year-old (n=196)**	16.08	9.36–27.61	0.000
**Not receiving raw meat (n=178)**	Reference		
**Receiving raw meat (n=913)**	2.46	1.58–3.82	0.000

Area under the receiver operating characteristic curve = 0.7587

S5G Table. The multivariable logistic regression model for *Toxoplasma gondii* seropositivity in 1121 cats of eight breeds, using the Siamese as the reference breed.

	Odds ratio	95% confidence interval	P-value
**Siamese (n=43)**	Reference		
**Birman (n=281)**	1.62	0.77–3.42	0.205
**British Shorthair (n=107)**	1.32	0.58–3.02	0.514
**Burmese (n=85)**	0.39	0.15–0.99	0.047
**Korat (n=114)**	0.79	0.33–1.86	0.593
**Norwegian Forest Cat (n=343)**	1.81	0.87–3.79	0.113
**Ocicat (n=88)**	1.66	0.71–3.86	0.239
**Persian (n=60)**	2.72	1.10–6.74	0.031
**< 1 year old (n=165)**	Reference		
**1-year-old (n=286)**	1.74	1.07–2.83	0.025
**2-year-old (n=159)**	2.02	1.19–3.43	0.009
**3-year-old (n=103)**	4.19	2.36–7.45	0.000
**4–6-year-old (n=190)**	4.91	2.97–8.13	0.000
**7–19-year-old (n=196)**	16.08	9.36–27.61	0.000
**Not receiving raw meat (n=178)**	Reference		
**Receiving raw meat (n=913)**	2.46	1.58–3.82	0.000

Area under the receiver operating characteristic curve = 0.7587
